# Protective efficacy of a recombinant adenovirus expressing novel dual F and HN proteins of bovine parainfluenza virus type 3

**DOI:** 10.1186/s13567-024-01400-z

**Published:** 2024-11-07

**Authors:** Jiaqi Zhang, Jinbo Wu, Qing Zhu, Xiangyue Huang, Zhaohui Zhang, Chenxi Zhu, Gunan Deng, Ajia Ake, Yuanzhen Ma, Chunsai He, Rui Guo, Hua Yue, Lan Lan, Bin Zhang

**Affiliations:** 1 College of Animal and Veterinary Sciences, Southwest Minzu University, Chengdu, 610041 China; 2 Key Laboratory of Ministry of Education and Sichuan Province for Qinghai-Tibetan Plateau Animal Genetic Resource Reservation and Utilization, Chengdu, 610041 China; 3 Animal Husbandry Science Institute of ABa Autonomous Prefecture, Hongyuan, 624400 China; 4 Center for Animal Disease Control and Prevention, Ganzi Tibetan Autonomous Prefectue, Kangding, 626000 China; 5 Animal Husbandry Science Institute of Ganzi Tibetan Autonomous Prefecture, Kangding, 626000 China

**Keywords:** Bovine parainfluenza virus type 3, adenovirus vector vaccine, F and HN proteins, protection efficacy

## Abstract

Bovine parainfluenza virus type 3 (BPIV3) is a viral respiratory pathogen that infects cattle and causes significant economic losses. We generated a recombinant adenovirus called rHAd5-F + HN by expressing the fusion (F) and hemagglutinin-neuraminidase (HN) glycoprotein of BPIV3 using the human adenovirus serotype 5 (rHAd5). We evaluated its effects on humoral and cellular immune responses in mice (*n* = 45) and calves (*n* = 9). Serum antibody responses were assessed by enzyme-linked immunosorbent assay (ELISA), hemagglutination inhibition (HI), and neutralising antibodies (NAb). After boosting immunity with rHAd5-F + HN, mice produced significantly higher levels of antibodies against the BPIV3 genotype A and genotype C strains. The production of antibodies exceeded those produced by adenoviruses rHAd5-F and rHAd5-HN, which express the F and HN glycoprotein, respectively. The percentages of splenic CD3^+^/CD8^+^T lymphocytes and IL-4^+^ cytokines in rHAd5-F + HN mice were considerably higher than those in the control group. Mice immunised with rHAd5-F + HN exhibited much lower viral loads in the lungs and tracheas compared to the control group. Additionally, the lungs of mice vaccinated with rHAd5-F + HN showed no notable histopathological changes. On the other hand, rHAd5-F + HN produced a humoral immune response in calves. Following the booster intramuscular injection with the rHAd5-F + HN, the serum antibody levels against BPIV3 genotype C strain were 1:20 452, 1:1024, and 1:426 in calves, as detected by ELISA, HI, and NAb, respectively. The HI and NAb levels against the BPIV3 genotype A strain were 1:213 and 1:85 in calves, respectively. These results indicate that rHAd5-F + HN effectively induced immunity against BPIV3 infection.

## Introduction

Bovine parainfluenza virus type 3 (BPIV3) is a respiratory tract pathogen from the genus *Respirovirus* within the *Paramyxoviridae* family [1]. BPIV3 commonly coexists with bovine coronavirus (BCoV), bovine herpesvirus type 1 (BHV-1), bovine respiratory syncytial virus (BRSV), bovine viral diarrhoea virus (BVDV), and *Mycoplasma bovis* [2]. These co-infections, coupled with the potential for secondary bacterial infections such as *Pasteurella multocida*, *Histophilus somni*, and *Mannheimia haemolytica*, play a significant role in the development of Bovine respiratory disease complex (BRDC) [3]. BRDC has cost the US beef cow-calf industry approximately $165 million annually and has also resulted in substantial economic losses in the cattle industry worldwide [4].

The genome of BPIV3 spans approximately 15,000 nucleotides, containing genes for six structural proteins (N, P, L, M, F, and HN) and three non-structural proteins (C, D, and V) [5]. BPIV3 is classified into three genotypes: A, B, and C [6]. The novel BPIV3 genotype C was first identified in China and has since spread globally [2, 4, 5, 7–9]. Epidemiological investigations have revealed that genotype C is the predominant strain in China [9]; however, no commercial vaccine is available in China [10]. Previous studies have shown a high BPIV3 genotype C transmissibility and a reduced cross-reactivity of genotype C sera to genotypes A and B viruses [11, 12]. Additionally, several studies indicate that cattle vaccinated with the BPIV3 A vaccine exhibit lower levels of neutralising antibodies against the BPIV3 C strain in serum than those against the BPIV3 A strain. This finding suggested a potential weakening in the cross-protective efficacy against genotype C [5, 8, 13, 14]. Thus, there is an urgent need for a safe and effective vaccine to manage epidemics caused by the BPIV3 genotype C strain.

As protective antigen in the *paramyxovirus* family, the fusion (F) protein and hemagglutinin-neuraminidase (HN) protein can stimulate the production of neutralising antibodies, making them ideal targets for developing genetic engineering vaccines [15–21]. A self-cleaving 2 A peptide between genes, facilitating the simultaneous expression of multiple genes [22], has proven pivotal in developing multi-gene expression vectors in recent years [23]. The self-cleaving peptide P2A, consisting of 22 amino acids, is located between two porcine teschovirus proteins and can efficiently cleave adjacent proteins through ribosomal skipping, with a high-efficiency rate of 99% [24]. This technique has been commonly used to construct multi-gene expression vectors [25]. Moreover, these vectors have been successfully employed in developing genetic engineering vaccines, including the recombinant adenovirus vectors [26, 27].

The replicating-defective recombinant human adenovirus type 5 (rHAd5) has been widely used as a vector in vaccine development due to its ability to induce strong cellular, humoral, and mucosal immune responses in animals [28, 29]. Many adenovirus vectors have been developed based on the F and HN proteins within the *Paramyxoviridae* family, showcasing their effective humoral and cellular immune responses [30, 31]. In a previous study, we successfully developed a recombinant adenovirus (rHAd5-F) that expressed the F protein of BPIV3 genotype C while exhibiting excellent immunogenicity and demonstrating significant protective potential in mice [21].

Furthermore, several studies indicate that human adenoviruses do not show antigenic cross-reactivity with the nine serotypes of adenovirus found in cattle [32]. Using human adenovirus as a vector may help reduce the risk of decreased immune effectiveness caused by pre-existing adenovirus antibodies in cattle. Our objective in this study was to develop a vaccine against BPIV3. Specifically, we developed a novel vaccine with enhanced immunogenicity: a recombinant adenovirus founded on rHAd5 with P2A added to enable the co-expression of the F and HN proteins.

## Materials and methods

### Experimental animals

For this experiment, we sourced specific pathogen-free (SPF) female BALB/c mice, aged 5–6 weeks and weighing 14–16 g each, from Chengdu Dossy Experimental Animals Co, Ltd. Commercially raised Holstein male calves, aged 2–3 months and weighing 90–100 kg each, were sourced from Sichuan Xuebao Dairy Group Company.

### Cell lines and viruses

The HEK293 cell containing the adenovirus E1 gene and Madin-Darby bovine kidney (MDBK) cell lines are preserved in our laboratory. BPIV3 genotype C (SMU-SC20) strain (GenBank: OM621819.1) [33] and BPIV3 genotype A (B15) strain (GenBank: PP025529.1) were isolated from yaks and water buffaloes suffering from BRDC, respectively. The titres of the virus were 1 × 10^6.5^ TCID_50_/mL and 1 × 10^6.45^ TCID_50_/mL, respectively [33]. The recombinant adenovirus rHAd5-WT, devoid of foreign genes, was engineered and maintained within our laboratory. The rHAd5-WT was obtained by co-transfecting HEK293 cells with the adenoviral shuttle plasmid (pDC316), which does not carry any exogenous genes, and the adenoviral backbone plasmid (pBHGloxΔE1,3Cre).

### Construction and characterisation of recombinant adenoviruses expressing BPIV3 F and HN proteins

The F and HN genes of BPIV3, derived from the SMU-SC20 strain, were codon-optimised using UpGene software. The self-cleaving peptide P2A was strategically inserted between the F and HN genes to enable the simultaneous expression of both genes in a concatenated form. The TAA stop codon at the end of the F gene was excised, and the P2A gene was ligated between the F and HN genes before gene synthesis (Sangon Biotech, Shanghai, China). The process of inserting the F and HN genes into the E1 gene of the recombinant adenovirus involved co-transfecting the adenoviral shuttle plasmid (pDC316) containing the target genes with the adenoviral backbone plasmid (pBHGloxΔE1,3Cre) into the HEK293 cells for homologous recombination. This procedure was followed with further analysis.

The specific transfection steps were as follows: HEK293 cells (10% calf serum and Dulbecco’s Modified Eagle Medium (DMEM)) were seeded in a six-well plate at a density of 7 × 10^5^ cells per well. After 24 h of culture, 3.2 µg of backbone plasmid (pBHGlox_E1, 3Cre) and 0.8 µg of shuttle plasmid containing the foreign gene (pDC316), were transfected using TurboFect transfection reagent, as per the manufacturer’s instructions. The cells were monitored daily, and a viral harvest was undertaken when most cells had detached.

Western blot (WB) and indirect immunofluorescence (IFA) techniques were performed to confirm the expression of the F and HN proteins in the recombinant adenoviruses. The specific methods were as previously described [21]. The primary antibodies in the WB and IFA were polyclonal mouse anti-BPIV3 whole virus sera prepared in our laboratory.

The rHAd5-HN and rHAd5-F + HN viruses were each passaged 12 times in succession. Passages 1, 3, 6, 9, and 12 were examined to evaluate the stability of the viruses. To extract the DNA genome of the recombinant adenovirus, 20 µL of the viruses were initially taken, and 2 µL of proteinase K was added. The mixture was digested at 50 °C for 30 min to release the viral genome. The released genome served as a template for amplification and insertion into the recombinant adenovirus gene. The HN and F + HN genes were amplified using pDC316 universal primers, followed by sequence alignment. The pDC316 universal primers were forward, 5′- ACGTGGGTATAAGAGGCG − 3′ and reverse, 5′- CGATGCTAGACGATCCAG − 3′.

### Chromatographic purification of recombinant adenoviruses

The recombinant adenoviruses were purified through anion exchange chromatography to enhance the purity of these viruses in this experiment. Recombinant adenoviruses were purified using two methods: Diamond Layer 700 BA, which employs anionic and hydrophobic interactions and molecular sieving, and Diamond Q Mustang, which involves ion exchange interactions. The HN and F + HN genes were amplified using the universal primers of pDC316. Following purification, the viral titre (TCID_50_ mL^−1^) of recombinant adenoviruses was determined in HEK293 cells using the Reed-Muench method.

To determine the viral growth kinetics of these recombinant adenoviruses, we collected cell culture supernatants every 12 h post-infection to determine the TCID_50_ at each time point.

### Immunisation and challenge

To study the effect of varying dosages on the immune response to recombinant adenovirus, we divided the recombinant adenovirus (rHAd5-F + HN, rHAd5-F, rHAd5-HN) into high- and low-titre groups. The SPF BALB/c female mice were intramuscularly injected into their thigh muscles. The intramuscular (IM) injection volume was 200 µL, with a titre of (1 × 10^6^ TCID_50_ mL^−1^) for the high-titre group and (1 × 10^5^ TCID_50_ mL^−1^) for the low-titre group, as described in reference [34]. The inactivated BPIV3 were administered intramuscularly at 200 µL (1 × 10^6.5^ TCID_50_ mL^−1^). The phosphate-buffered saline (PBS) and the rHAd5-WT control group were included. All mice were given a second immunisation booster of the same dose two weeks after the initial immunisation. Serum samples were collected from the immunised mice every week after the first immunisation to evaluate the humoral immune response of the recombinant adenoviruses in mice. The variations in immunoglobulin G (IgG) levels, HI antibody titres and NAb titres in the serum samples of mice were monitored, and statistical analysis was conducted 14 days after the second immunisation.

In the third week after the booster immunisation, all mice were challenged with 100 µL of the SMU-SC20 strain at a titre of 1 × 10^6.5^ TCID_50_ mL^−1^ to assess the vaccine’s protective efficacy. On day 7 post-infection (dpi), all mice were euthanised, and necropsy was performed. The tracheas and lungs of the mice were collected to measure the levels of BPIV3 mRNA. In addition, histopathological analysis was conducted on the lung tissues.

After nasal swabs were collected from the male Holstein calves, BVDV, BPIV3, BHV-1, and BCoV tests were performed. The results showed that all tests were negative. Before immunisation, we obtained sera from calves to test for HI antibodies and NAb against both BPIV3 genotype A and genotype C strains. Only seronegative calves were selected for the immunisation experiments, all of whom had received colostrum.

The rHAd5-F + HN was administered via IM injection for immunisation, with a total volume of 2 mL (1 × 10^6.4^ TCID_50_ mL^−1^). The inactivated BPIV3 was administered intramuscularly at 2 mL (1 × 10^6.5^ TCID_50_ mL^−1^), and the control calves were intramuscularly injected with PBS 2 mL. All calves received a booster immunisation at the same dose three weeks after the initial immunisation. The changes in IgG levels, HI antibody, and NAb titres were dynamically monitored. Statistical analysis was conducted using the data from bovine serum collected 14 days after the second immunisation.

### Indirect enzyme-linked immunosorbent assay (ELISA)

To detect BPIV3-specific IgG antibodies in serum samples from mice and calves, we coated a 96-well polyethylene microtiter plate with the inactivated SMU-SC20 strain and incubated it overnight at 4 °C. The secondary antibodies used were anti-mouse-HRP or anti-bovine-HRP (Millipore Sigma) at a dilution of 1:5000, respectively. The optical density of each well was determined using a microplate reader (Bio-Rad, Hercules, CA, USA) at 450 nm. The endpoint titre was the highest reciprocal serum dilution that yielded an absorbance ≥ 2.1-fold over negative control serum values. The specific methods were as previously described [21].

### BPIV3 hemagglutination inhibition (HI) assay

A hemagglutination assay was performed to determine the hemagglutination titre of the BPIV3 virus. The assay used a 96-well U-bottom plate with a 1% suspension of chicken red blood cells. Equal volumes (50 µL) of serially diluted virus isolates were mixed with the red blood cells, thoroughly agitated, and allowed to settle for 30 min at 37 °C. The endpoint was defined as the last well in which chicken red blood cells showed no signs of aggregation. The HI antibodies were measured using the methods previously described [18].

Serum samples were serially diluted two-fold (50 µL each) and were incubated at 37 °C for 1 h with an equal volume of diluent containing the hemagglutination units of either SMU-SC20 or B15 strains. The 50 µL of 1.0% chicken red blood cells was added, and the mixture was incubated at 37 °C for 1 h. The HI antibody titre was determined by identifying the highest serum dilution that completely inhibited hemagglutination. The serum samples of mice and calves were subjected to a HI assay to detect the presence of HI antibodies against the SMU-SC20 and B15 strains.

### BPIV3 neutralisation assay (NAb)

As previously described, the serum neutralisation test determined NAb titres against SMU-SC20 and B15 strains in mice and calves [21]. The serum was diluted two-fold, and 100 µL of the diluted sample was mixed with an equal volume of viral supernatant containing 200 TCID_50_/0.1 mL of either SMU-SC20 or B15 strains and then incubated. The highest serum dilution showing a cytopathic effect of more than 50% was chosen as the titre for virus-neutralising antibodies. The serum samples of mice and calves were analysed using a virus-neutralisation assay to ascertain the presence of NAb against the SMU-SC20 and B15 strains.

### Flow cytometry assays

To evaluate whether the recombinant adenovirus rHAd5-F + HN can stimulate cellular immune responses in mice, we isolated splenic lymphocytes from immunised mice (*n* = 5 in each group) three weeks after the immunisation boost. The isolated splenic lymphocytes were resuspended at a concentration of 5 × 10^6^ cells/mL in RPMI-1640. Subsequently, the cells were seeded into 6-well plates at 2 mL per well and stimulated with the inactivated SMU-SC20 strain (60 µg/mL) and ionomycin (1 µg/mL) for 12 h. After the incubation period, the cells were stained with a series of antibodies, including anti-CD3-FITC, anti-CD4-FITC, anti-CD8-PE/Cy7, anti-IFN-γ, and anti-IL-4 (Biolegend, USA) at 4 °C for 2 h. The percentage of positive cells was measured using the CytoFLEX cell analyser (Beckman, CA, USA).

### Quantitative reverse-transcription polymerase chain reaction (RT-PCR)

As previously mentioned, our laboratory developed a real-time RT-PCR method to quantify the viral loads of BPIV3 in mice tissues. This was achieved using primers that bind the P gene [21]. P gene-specific primers were (forward, 5′-ARAGGACACAGAAGAGAGCACT-3′; reverse, 5′-TRGCCACACATACAACTCTCT-3′) and probe (5′-FAM-TTACAGAAAGGGCGATTACATTATTACAGA-BHQ1-3′). The RT-PCR method had an assay sensitivity of 1.0 × 102 copies/µL. The virus loads were calculated as y = − 3.203x + 45.76.

### Histopathological examination

The lung tissues from mice were stained with Haematoxylin and Eosin and observed for pathological changes using an optical microscope (Leica DM4000B, Wetzlar, Germany).

### Statistical analysis

The data were analysed and plotted using GraphPad Prism software (version 10.1.2). ELISA, HI, NAb, T-cell data. The viral burden were analysed using one-way (ANOVA) with Bonferroni multiple comparisons. Data are expressed as the mean with standard error (SEM). Statistical significance was determined with a threshold of (*p* < 0.05). Antibody titre and virus copy number data were log-transformed before analysis. All performed experiments were repeated at least thrice.

## Results

### Construction and identification of rHAd5-HN and rHAd5-F + HN

To ensure experimental consistency, we simultaneously identified and purified three types of adenoviruses. The F genes span a length of 1623 base pairs (bp), encoding a total of 540 amino acids (aa). The entire coding sequence of the HN genes was 1719 bp in length, corresponding to 573 aa. The P2A gene was found to be 84 bp in length, encoding a protein consisting of 28 aa. This protein was subsequently inserted between the F and HN genes, allowing for the translation of a protein that could be cleaved into two distinct, independent proteins.

**Figure 1:**
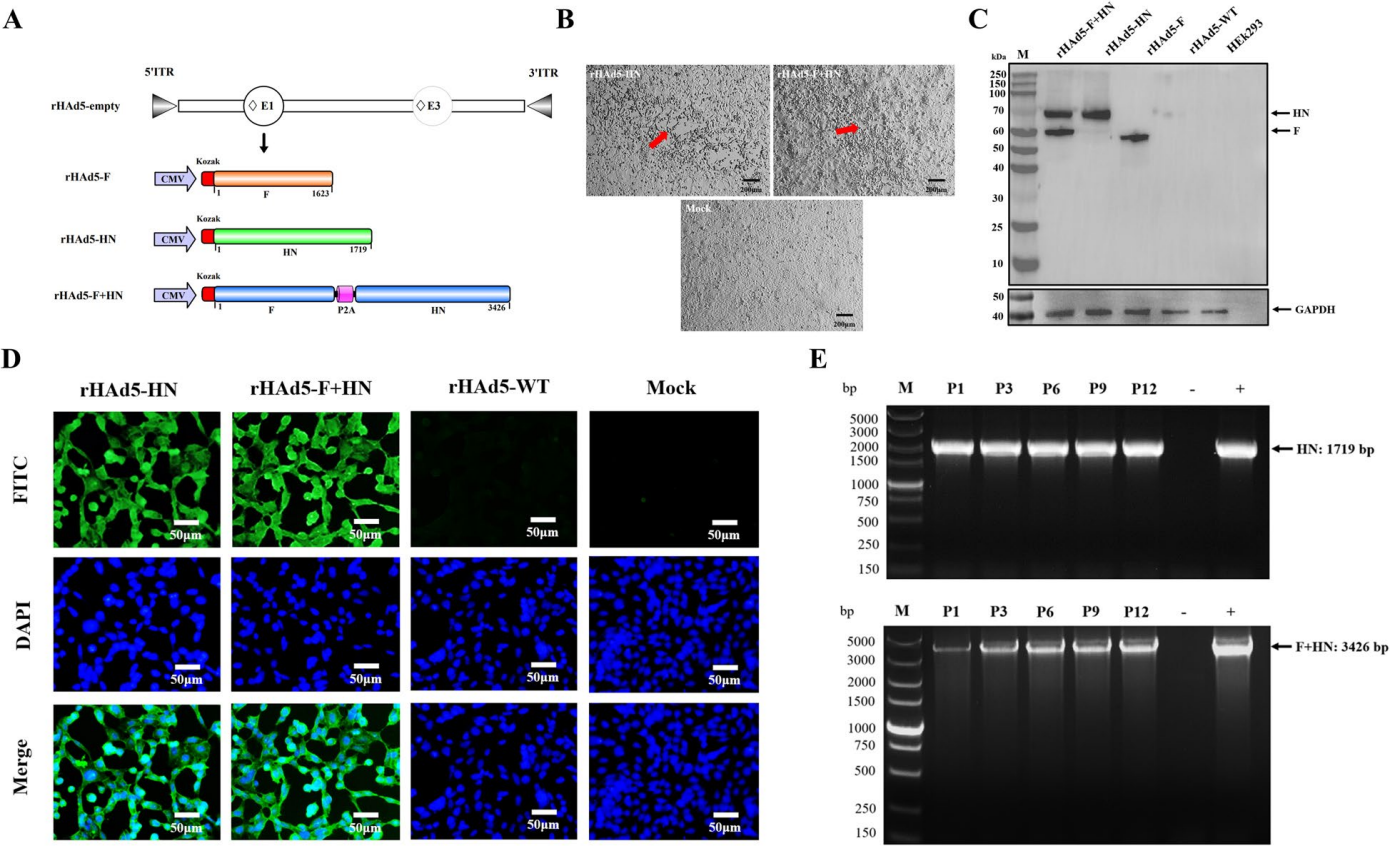
**Construction and identification of recombinant adenoviruses**. **A** Schematic diagram of the genomes of HAd5-F, rHAd5-HN and rHAd5-F + HN; **B** the cytopathic effect (CPE) induced by rHAd5-HN and rHAd5-F + HN in HEK293 cells is indicated by red arrows in the figure; **C** Western blot analysis of F and HN protein expression in HEK293 cells infected with HAd5-F + HN, rHAd5-HN, rHAd5-F, and rHAd5-WT; **D** immunofluorescence analysis of HAd5-HN, rHAd5-F + HN and rHAd5-WT; **E** PCR detection of rHAd5-HN and rHAd5-F + HN, “P” means passage. “+; −” means positive or negative control.

The schematic diagram of the recombinant adenovirus construct is shown in Figure 1A. The infection of recombinant adenoviruses resulted in cytopathic effects that were consistent with the formation of adenoviruses within the cells (Figure 1B). The results of the WB experiment indicated the expression of F and HN proteins (Figure 1C), which was validated through an immunofluorescence assay, where specific green fluorescence was observed under a fluorescence microscope (Figure 1D). Based on these findings, the recombinant adenoviruses successfully expressed the F and HN proteins in HEK293 cells.

The PCR analysis of the serially passaged recombinant adenoviruses revealed the detectable presence of HN and F + HN genes at every passage. Sequencing confirmed the accuracy of the results (Figure 1E), denoting the genetic stability of the recombinant adenoviruses.

### Recombinant adenoviruses purified by chromatography

**Figure 2:**
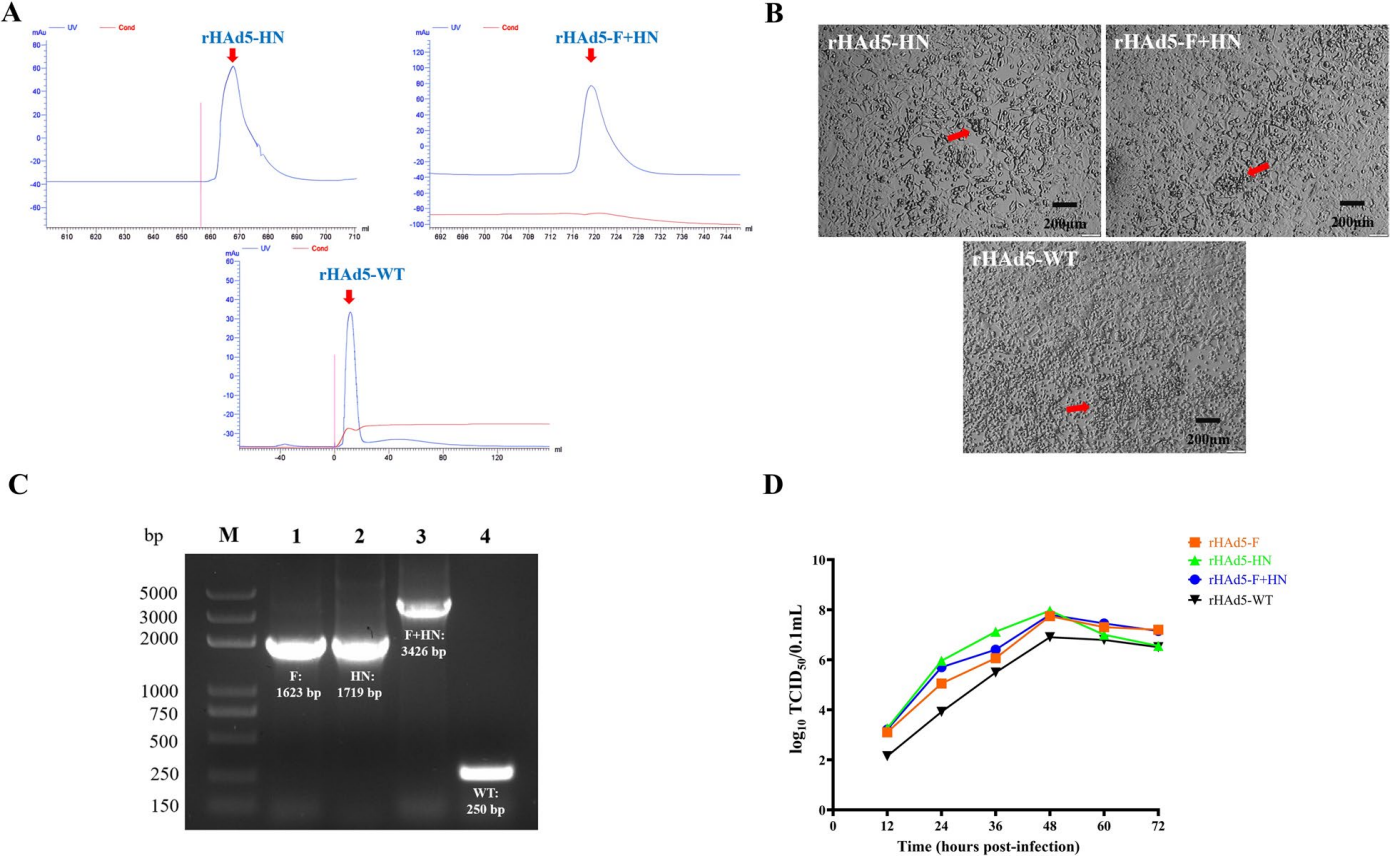
**Chromatographic purification and identification of recombinant adenoviruses**. **A** The collection of chromatographically purified products from recombinant adenoviruses; **B** purified rHAd5-HN, rHAd5-F + HN, and rHAd5-WT on adenovirus-related cytopathic effects in HEK293 cells is indicated by red arrows in the figure; **C** PCR detection of purified rHAd5-F, rHAd5-HN, rHAd5-F + HN and rHAd5-WT; **D** growth curves of purified HAd5-F, rHAd5-HN, rHAd5-F + HN and rHAd5-WT viruses post purification.

The purified liquid containing the recombinant adenoviruses was collected at the point when a peak appeared on the UV curve (Figure 2A). The purified recombinant adenoviruses were then used to infect HEK293 cells to assess their remaining infectivity (Figure 2B). The presence of exogenous genes in the purified recombinant adenoviruses was verified by conducting PCR analysis (Figure 2C). The TCID_50_ values of the purified recombinant adenoviruses, namely rHAd5-F, rHAd5-HN, rHAd5-F + HN and rHAd5-WT, were calculated using the Reed-Muench method and were 1 × 10^6.06^ TCID_50_/0.2 mL^−1^, 1 × 10^7.12^ TCID_50_/0.2 mL^−1^, 1 × 10^6.40^ TCID_50_/0.2 mL^−1^, and 1 × 10^5.49^ TCID_50_/0.2 mL^−1^, respectively (Figure 2D). These results signified that the recombinant adenoviruses were purified.

### Recombinant adenoviruses induced humoral immune responses in mice

**Figure 3:**
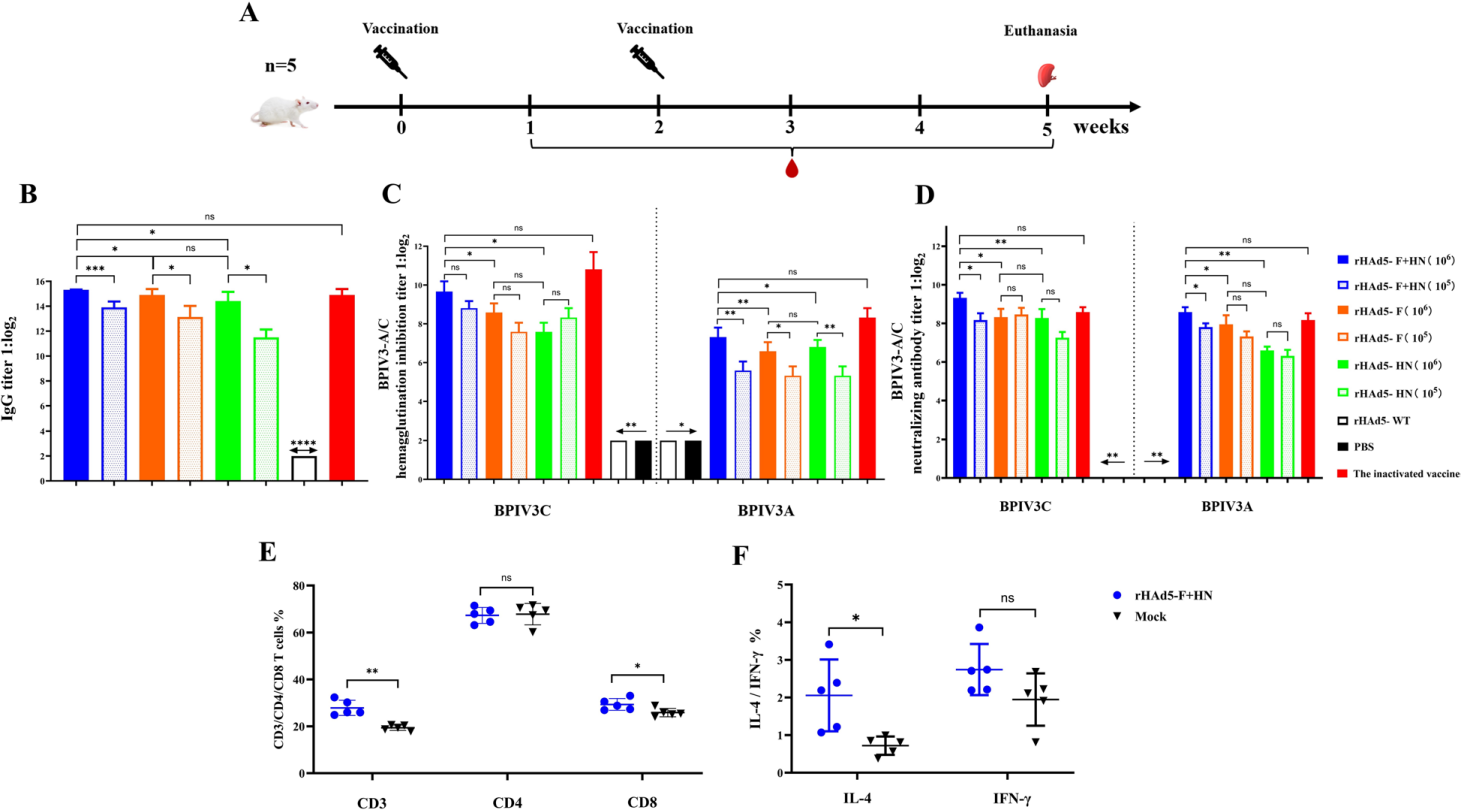
**Immunogenicity assessment of recombinant adenoviruses in mice**. **A** Immunisation schematic diagram; **B** IgG antibody titers against the BPIV3 genotype C strain in serum; **C** HI antibody titers against the BPIV3 genotype A and genotype C strains in serum; **D** NAb titers against the BPIV3 genotype A and genotype C strains in serum; **E** The percentages of CD3^+^, CD4^+^ and CD8^+^T lymphocytes in spleens of mice immunised with HAd5-F + HN; **F** The percentages of IFN-γ^+^/IL-4^+^ cytokine in spleens of mice immunised with HAd5-F + HN.

SPF BALB/c female mice were randomly assigned to nine groups, with five per group (Table 1). The immunisation  of mice are shown in Figure 3A. During the peak period of 14 days after the second immunisation, the comparison of IgG antibody titres among different groups showed that the high-dose groups (rHAd5-F, rHAd5-HN, and rHAd5-F + HN) of recombinant adenoviruses had significantly higher antibody titres compared to the low-dose groups rHAd5-F (*p* < 0.05), rHAd5-HN (*p* < 0.05), and rHAd5-F + HN (*p* < 0.001) (Figure 3B). The average IgG antibodies titre in the high-dose rHAd5-F + HN group reached a peak of 1:40 906, which was significantly higher than the titres observed in the high-dose rHAd5-F group (1:28 924, *p* < 0.05) and the high-dose rHAd5-HN group (1:17 198, *p* < 0.05). The average IgG antibody titre in the BPIV3 inactivated vaccine group was 1:28 526, which was lower than the high-dose group rHAd5-F + HN. However, the statistical analysis showed no significant difference (ns, *p* ≥ 0.05).

**Table 1:** **Types and dosages of vaccines for immunised mice**.

Groups	Types of vaccines	Dose of immunity	The number
A	rHAd5-F + HN (high dose)	200 µL (1 × 10^6^ TCID_50_ mL^−1^)	5
B	rHAd5-F (high dose)	200 µL (1 × 10^6^ TCID_50_ mL^−1^)	5
C	rHAd5-HN (high dose)	200 µL (1 × 10^6^ TCID_50_ mL^−1^)	5
D	rHAd5-WT	200 µL (1 × 10^6^ TCID_50_ mL^−1^)	5
E	rHAd5-F + HN (low dose)	200 µL (1 × 10^5^ TCID_50_ mL^−1^)	5
F	rHAd5-F (low dose)	200 µL (1 × 10^5^ TCID_50_ mL^−1^)	5
G	rHAd5-HN (low dose)	200 µL (1 × 10^5^ TCID_50_ mL^−1^)	5
H	Inactivated BPIV3	200 µL (1 × 10^6.5^ TCID_50_ mL^−1^)	5
I	PBS	200 µL	5

In the high-dose rHAd5-F + HN group, the HI titres in the serum were 1:813 (genotype C) and 1:160 (genotype A) (Figure 3C). These titres were significantly higher than those of the high-dose rHAd5-F group (1:384) (genotype C) (*p* < 0.05) / (1:96) (genotype A) (*p* < 0.01) and the high-dose rHAd5-HN group (1:192) (genotype C) (*p* < 0.05) / (1:112) (genotype A) (*p* < 0.05). The average HI antibodies titre in the BPIV3 inactivated vaccine group were 1:1,792 (genotype C) and 1:320 (genotype A). This outcome is greater than that of the high-dose rHAd5-F + HN group.

In the high-dose rHAd5-F + HN group, the NAb titres in the serum were 1: 640 (genotype C) and 1:384 (genotype A), a result that was significantly higher than the high-dose rHAd5-F group (1:320) (genotype C) (*p* < 0.05)/(1:247) (genotype A) (*p* < 0.05) and the high-dose rHAd5-HN group (1:301) (genotype C) (*p* < 0.01)/(1:96.5) (genotype A) (*p* < 0.01) (Figure 3D). The average NAb titres in the BPIV3 inactivated vaccine group were 1:488 (genotype C) and 1:288 (genotype A). These outcomes were lower than that of the high-dose rHAd5-F + HN group.

The results suggested that the rHAd5-F + HN group could generate higher IgG antibody titres and robust humoral immune responses compared to the rHAd5-F group, rHAd5-HN group, and inactivated BPIV3 group. Thus, the rHAd5-F + HN could induce the production of high-titre HI and NAb in mice and have a neutralising effect on different genotypes of BPIV3 strains.

### Recombinant adenovirus-induced cellular immune responses in mice

The percentages of CD3^+^ T and CD8^+^ T lymphocytes and the IL-4 of cytokine in the rHAd5-F + HN group were significantly higher compared to the PBS group (*p* < 0.01)/(*p* < 0.05)/(*p* < 0.05). Figure 3E, F shows the cellular immune response of rHAd5-F + HN in mice. These results indicated that rHAd5-F + HN had a powerful immunogenic effect, inducing a greater number of mature DCs in the spleen while enhancing T lymphocyte proliferation.

### Recombinant adenoviruses protected mice against BPIV3 infection

**Figure 4:**
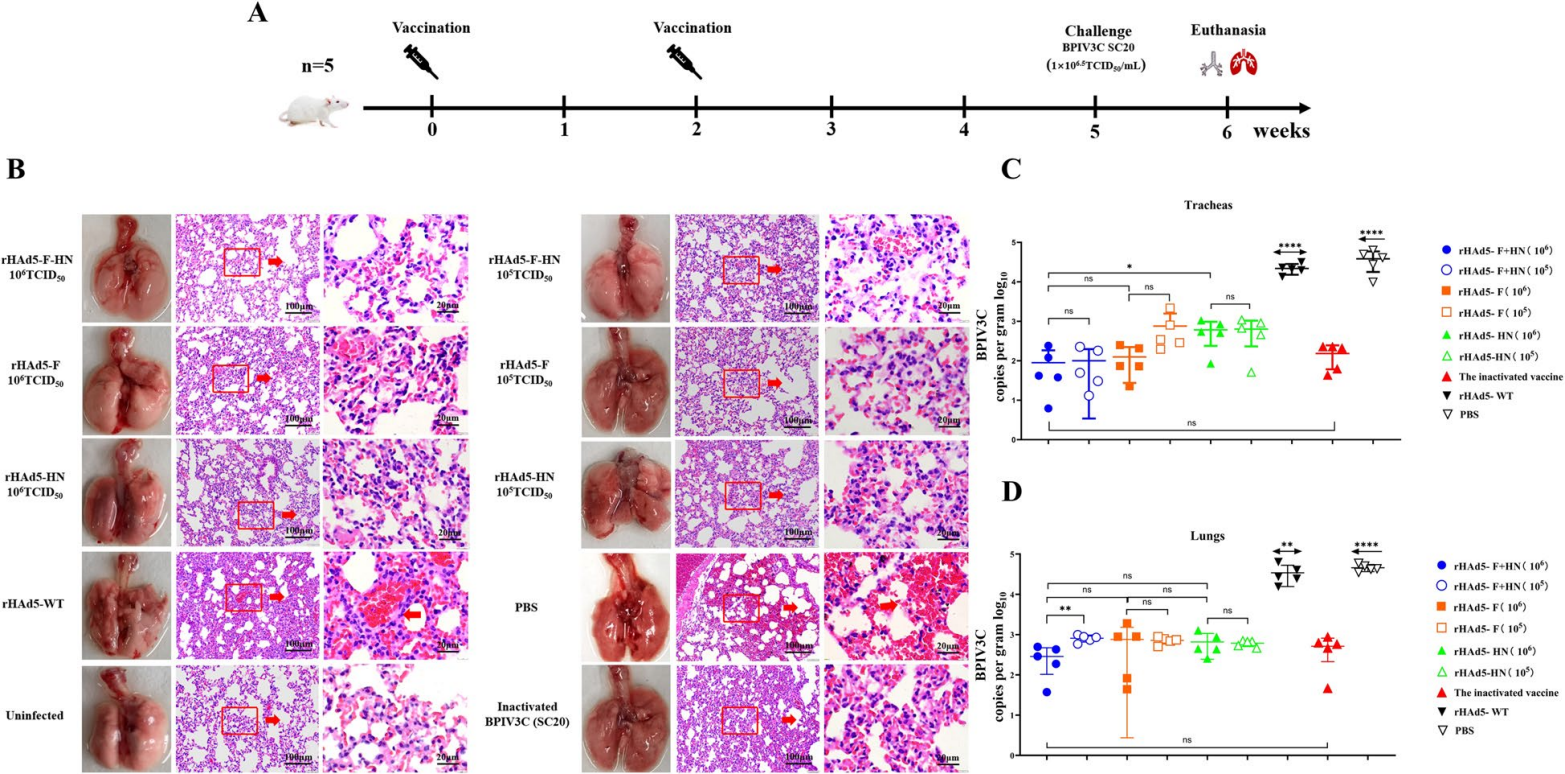
**Assessment of the protective efficacy of recombinant adenoviruses in mice**. **A** Immunisation and infection schematic diagram; **B** histopathological analysis of BPIV3 SC20 strain-infected mice; **C**, **D** relative mRNA levels of BPIV3 in tracheas and lungs of mice determined by qRT-PCR.

The challenge of mice are shown in Figure 4A. The viral loads in the tracheas of the mice that were injected with the high-dose rHAd5-F + HN group were significantly lower than those in the high-dose rHAd5-HN group (*p* < 0.05) (Figures 4C and D). However, there were no significant differences in viral load in the tracheas and lungs between the rHAd5-F + HN high-dose and the low-dose groups (*p* > 0.05).

We visually assessed and performed a histopathological analysis of the lungs after exposing them to the SMU-SC20 strain (Figure 4B). Microscopy and visual assessment showed no pathological changes in the lungs of mice in the high-dose groups (such as rHAd5-F, rHAd5-HN, and rHAd5-F + HN), the uninfected groups, and the inactivated BPIV3 groups. However, there were visible pathological changes, such as swelling and bleeding. Microscopic analysis showed varying degrees of alveolar epithelial hyperplasia, interalveolar capillary hyperaemia, and infiltration of lymphocytes and red blood cells in the alveolar septum and bronchioles in the lungs of mice from both the PBS and rHAd5-WT groups. In the low-dose groups of rHAd5-F and rHAd5-HN, the lungs also showed varying degrees of mild haemorrhage, congestion, thickening of alveolar septa, and infiltration of red blood cells. These findings suggest that mice immunised with a low dose of rHAd5-F and rHAd5-HN experienced varying degrees of lung tissue damage.

### Recombinant adenovirus-induced humoral immune responses in calves

**Figure 5:**
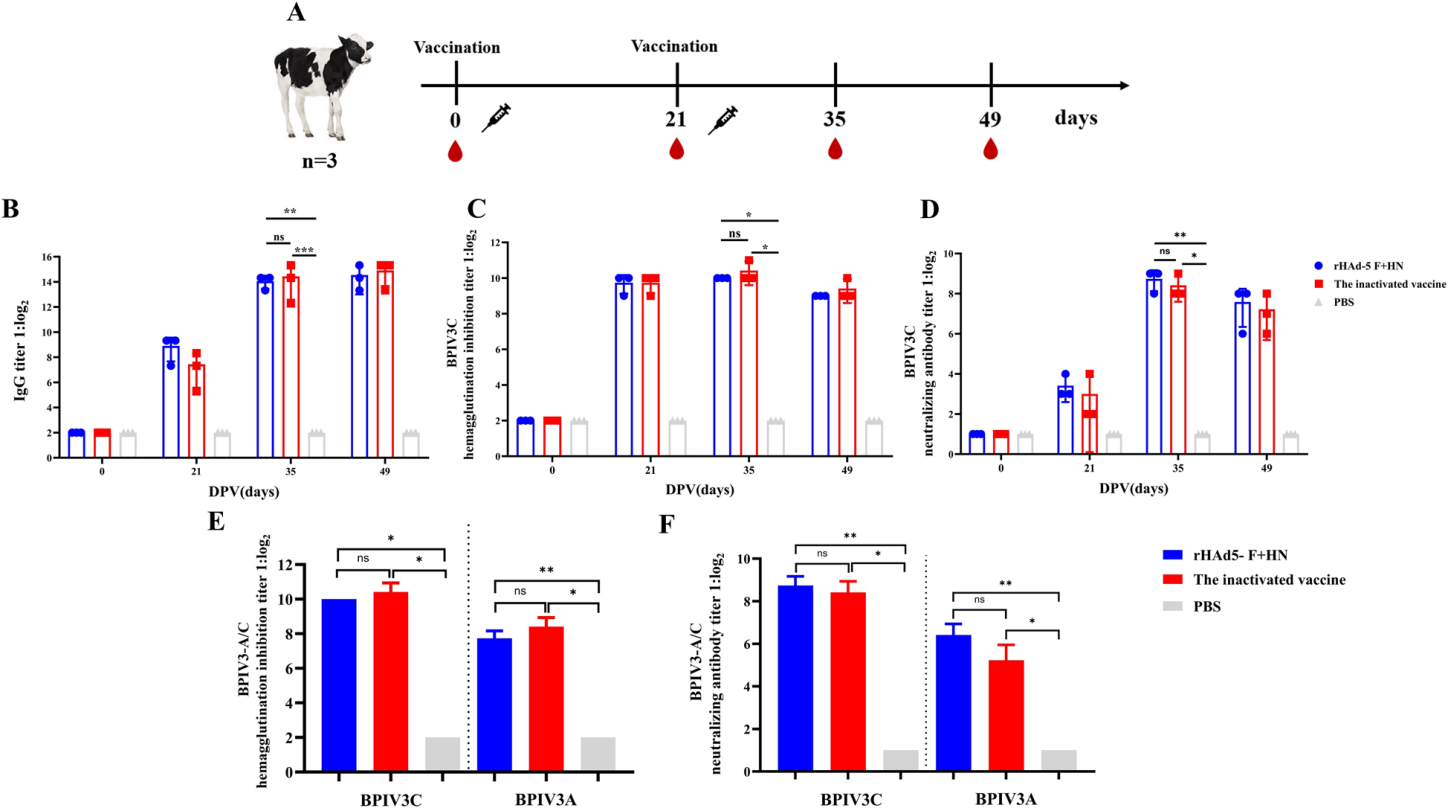
**Assessment of the immunogenicity of recombinant adenovirus in calves**. **A** Immunisation schematic diagram; **B** changes in IgG levels against the BPIV3 genotype C strain during the immunisation in serum; **C** changes in HI levels against the BPIV3 genotype C strain during the immunisation in serum; **D** changes in NAb levels against the BPIV3 genotype C strain during the immunisation in serum; **E** comparison of HI antibody titers against the BPIV3 genotype A and genotype C strains in serum; **F** comparison of NAb titers against the BPIV3 genotype A and genotype C strains in serum.

Nine male Holstein calves raised for commercial purposes were randomly divided into three groups, each consisting of three calves (Table 2). Serum samples were collected from immunised calves every two weeks after the second immunisation to evaluate the humoral immune response of the rHAd5-F + HN in calves (Figure 5A). After the second immunisation, the rHAd5-F + HN immunisation group exhibited an average IgG antibody titre of 1:20,452 at 26 days (Figure 5B). This result was significantly higher than that in the PBS group (*p* < 0.01). However, no significant difference was observed between the rHAd5-F + HN immunisation group and the BPIV3 inactivated vaccine group (ns, *p* ≥ 0.05).

**Table 2:** **Types and dosages of vaccines for immunised calves**

Groups	Types of vaccines	Dose of immunity	The number
A	rHAd5-F + HN	2 mL (1 × 10^6^ TCID_50_ mL^−1^)	3
B	The inactivated BPIV3	2 mL (1 × 10^6.5^ TCID_50_ mL^−1^)	3
C	PBS	2 mL	3

In the rHAd5-F + HN group, the average HI titres in the serum were 1:1024 (genotype C) and 1:213 (genotype A) (Figures 5C and E). This outcome was significantly higher than the PBS group (*p* < 0.05). The average HI titre in the BPIV3 inactivated vaccine group was 1:1,365 (genotype C) and 1:314 (genotype A), higher than the rHAd5-F + HN group. However, the two groups had no significant differences (ns, *p* ≥ 0.05).

In the rHAd5-F + HN group, the NAb titres in the serum were 1:426 (genotype C) and 1:85 (genotype A) (Figures 5D and F). This outcome was significantly higher than the PBS group (*p* < 0.01). The average NAb titre in the BPIV3 inactivated vaccine group was 1:341 (genotype C) and 1:37 (genotype A). This outcome was lower than the rHAd5-F + HN group. Again, the two groups had no significant differences (ns, *p* ≥ 0.05).

The results indicate that the recombinant adenovirus rHAd5-F + HN can stimulate the production of high levels of IgG, NAb, and HI antibodies in calves. Additionally, the virus has a neutralising effect on different genotypes of BPIV3 strains.

## Discussion

Bovine parainfluenza virus type 3 is one of the most significant respiratory pathogens in the development of BRDC, which can result in substantial economic losses in the cattle industry. Generally, vaccination is widely recognised as the most effective approach to prevent this disease [35]. However, despite this approach, no commercially available vaccines currently target this pathogen in China [1].

The purpose of this study was to create a potential vaccine through the rHAd5 system, which allows for the simultaneous expression of both F and HN proteins of BPIV3 using the P2A peptide. According to our WB analysis, the F and HN proteins could be completely cleaved and demonstrated strong biological activity. Additionally, the study confirmed the feasibility and advantages of using P2A to construct a polygene expression adenovirus vector. It also provided a practical reference for designing a BRDC multi-gene engineering vaccine.

Interestingly, the human respiratory syncytial virus (RSV), belonging to a member of the genus *Orthopneumovirus* in the *Pneumoviridae* family, was found to contain virus-like particles (VLPs) with both glycoproteins. These VLPs, when tested in a cotton rat model [36], exhibited superior immunogenicity compared to VLPs containing only the F or G protein. Moreover, the results indicate that the presence of the G protein in VLPs influenced the conformation of the F protein. This influence significantly elevated NAb levels and provided greater protection for cotton rats against RSV infection [36].

Previous studies have shown that both the G protein in RSV and the HN protein in BPIV3 are primary transmembrane surface glycoproteins of the virus. They are located in the same coding region of the viral genome and play similar roles in viral infection and entry [37, 38]. This study produced comparable findings, suggesting that the HN and F proteins could boost antibody levels and protective efficacy. Despite previous studies demonstrating that the two proteins could enhance their immunogenic effects [19], how they collaborate to augment their immunogenicity remains unclear.

In this study, recombinant adenovirus resulted in a strong humoral immune response in mice. The rHAd5-F + HN induced a significantly higher titre of IgG antibodies in mice than the rHAd5-F and rHAd5-HN; previous studies obtained similar results [19, 20]. Previous studies have also demonstrated that immunisation with rHAd5-F and rHAd5-HN in cotton mice could stimulate the production of HI and NAb antibodies against the BPIV3 genotype A sf4 strain [19]. These findings suggest that the rHAd5-F + HN vaccine induced cross-neutralising titres in BALB/c mice against two different genotypes of BPIV3. High titres were recorded, indicating that this vaccine may be potentially superior to those used in previous studies [19]. The reason for this outcome may be the influence of pre-existing immunity [39, 40]. Earlier preparations involved recombinant adenoviruses expressing F and HN proteins separately, requiring separate immunisations [19]. Consequently, an increased number of immunisations would significantly affect the immune efficacy of the adenovirus vector vaccine [39].

Other studies found that vaccines induce a cellular immune response critical for defending against respiratory viruses [41–43]. Similarly, we observed that rHAd5-F + HN significantly enhanced the proliferation of CD3^+^/CD8^+^ T lymphocytes and increased IL-4 production in mice. Therefore, these findings indicate that rHAd5-F + HN effectively induced a cellular immune response.

BALB/c mice are widely acknowledged as an optimal animal model for studying respiratory tract infections caused by BPIV3. Therefore, employing this model for virus challenge studies was essential in evaluating the protective efficacy of vaccines [18]. In this study, we employed BALB/c mice as a representative model to assess the immune protection conferred by the recombinant adenovirus against SMU-SC20 strain infection. Thus, the results indicate that the rHAd5-F + HN can potentially protect mice from BPIV3 genotype C infection.

The key indicators for determining whether a recombinant adenovirus candidate vaccine can be commercialised are the immunisation of cattle and the evaluation of its safety and immunological effects. However, there is limited research on genetic engineering vaccines developed for BPIV3, domestically and internationally, and on their immunological studies in calves. Haanes et al. utilised an insect cell baculovirus expression system to develop a subunit vaccine, HN-Baculo, incorporating the BPIV3 HN protein. Their study showed that calves immunised with HN-Baculo had elevated levels of a specific NAb serum and HI antibodies. The levels of NAb and HI antibodies induced in calves by the rHAd5-F + HN in this study were higher than those observed in other studies [44].

Additionally, the results of our study show that the rHAd5-F + HN vaccine candidate proved safe for calves and generated high levels of Nab and HI antibodies against BPIV3 genotypes A and C. However, this study has limitations due to the absence of challenge studies in calves to assess protective immunity. Therefore, there is insufficient data to comprehensively analyse the influence of gender, age, and weight on immune outcomes. In future evaluations of the vaccine’s immunogenicity, more cattle herds will be used to verify the vaccine’s ability to provide immunity and protect against pathogens.

In conclusion, we successfully constructed a candidate vaccine rHAd5-F + HN for BPIV3 and tested its humoral and cellular immunity in mice. The mice and calves immunised with rHAd5-F + HN generated immune responses against BPIV3 genotypes A and C. After a challenge, the mice immunised with the rHAd5-F + HN showed reduced lung pathological damage. This study presents the first evidence that simultaneous expression of the HN and F proteins of BPIV3 enhances its immune efficacy, thus laying a theoretical foundation for the future design of novel vaccines targeting BPIV3.
